# Predictors of gestational weight gain in western India: Findings from a longitudinal study across rural and urban cohorts

**DOI:** 10.1371/journal.pone.0328081

**Published:** 2025-07-28

**Authors:** Mugdha Deshpande, Neha Kajale, Nikhil Shah, Ketan Gondhalekar, Vivek Patwardhan, Anagha Pai Raiturker, Sanjay Gupte, Leena Patankar, Jasmin Bhawra, Anuradha Khadilkar, Tarun Reddy Katapally

**Affiliations:** 1 Department of Growth and Pediatric Endocrinology, Hirabai Cowasji Jehangir Medical Research Institute, Jehangir Hospital, Pune, Maharashtra, India; 2 Department of Health Sciences, Savitribai Phule Pune University, Pune, Maharashtra, India; 3 Department of Paediatric Endocrinology, MRR Children’s Hospital, Thane, Maharashtra, India; 4 Department of Obstetrics and Gynecology, Pai Raiturker Clinic, Pune, Maharashtra, India; 5 Gupte Hospital and Center for Research in Reproduction and Green Array Genomic Research and Solutions, Pune, Maharashtra, India; 6 Department of Obstetrics and Gynecology, Patankar hospital, Pune, Maharashtra, India; 7 School of Occupational and Public Health, Toronto Metropolitan University, Toronto, Canada; 8 DEPtH Lab, School of Health Studies, Faculty of Health Sciences, Western University, London, Ontario, Canada; 9 Department of Epidemiology and Biostatistics, Schulich School of Medicine and Dentistry, Western University, London, Ontario, Canada; 10 Children’s Health Research Institute, Lawson Health Research Institute, London, Ontario, Canada; University of Tennessee Knoxville, UNITED STATES OF AMERICA

## Abstract

**Background:**

Gestational weight gain-GWG is an important predictor of neonatal growth. However, there is dearth of literature from rural and urban India depicting longitudinal patterns and determinants of GWG. To address this gap, our objectives were to study longitudinal patterns and predictors of GWG in pregnant women residing in rural and urban areas in and around Pune city, Maharashtra, India and to compare them with pre-existing guidelines provided by IOM, 2009.

**Methods:**

This study enrolled 268(134-rural and 134-urban) healthy singleton pregnant women attending antenatal care centers in and around Pune, India between August 2020-September 2023. Participants were measured for anthropometry and interviewed for socioeconomic status, diet, physical activity, sleep quality, and prenatal distress once in each trimester. Pre-gestational weight status was calculated using WHO, Asian-Pacific, and South Asian BMI cut-points. GWG was estimated using IOM, 2009 guidelines.

**Findings:**

The observed mean GWG was 10.9 ± 4.2 kg(rural:9.9 ± 3.7, urban:11.9 ± 4.5). 61.2% of rural and 30% of urban underweight pregnant women did not gain adequate weight. 11.8% of rural and 57.3% of urban pregnant women with overweight or obese BMI exceeded recommended guideliness. Key predictors of inadequate GWG in second and third trimesters were low socio-economic status, parity, underweight pre-gestational BMI, prenatal distress, and poor sleep. The primary predictor of excessive GWG was overweight or obese pre-gestational BMI. These findings were consistent across all BMI classifications.

**Conclusion:**

Our findings indicate that urban underweight pregnant women gained significantly higher weight. There was health disparity between rural and urban pregnant women that needs to be addressed to improve health of pregnant women. We have identified important modifiable factors such as dietary intake, physical activity, etc. to ensure optimal GWG which can inform public health policies. Further research is needed to assess whether context-specific GWG recommendations would be beneficial as our study is based on single geographical location and timeframe.

## Introduction

Pregnancy is characterized by physical and physiological changes that occur to accommodate the growing needs of the fetus and to provide fat stores for childbirth and lactation [1]. Maternal health is a primary predictor for neonatal health and well-being, with good maternal health reducing the risk of chronic diseases. The impacts of maternal health on children’s health are especially critical in developing countries of the global south like India, where indicators such as neonatal mortality rate are still high (29.5%). Research has shown that there are significant differences in the percentage prevalence of neonatal mortality rate in India across rural (27.5%) and urban (18%) locations (National Family Health Survery-5) [2]. Among the common indicators of maternal health, gestational weight gain (GWG) is a modifiable factor of maternal health that plays a key role in the optimal growth of a child in-utero and at birth [3].

GWG is an indicator that estimates if adequate maternal gains have occurred. Achieving appropriate GWG is a significant contributor to fetal growth and maternal-fetal outcomes [4]. Studies suggest that trimester-specific patterns of GWG need to be studied as they are independently associated with fetal growth outcomes [5,6]. Inadequate or excessive GWG is known to cause severe perinatal complications, such as low-birthweight, small-for-gestational-age infant, or preterm birth for the fetus, as well as the need for a caesarean section, macrosomia, gestational diabetes mellitus, and difficulty in initiating breastfeeding in the mother, etc. [7,8]. To avoid these adverse maternal and fetal outcomes, in 2009, the IOM updated the guidelines for appropriate GWG according to various pre-gestational BMI categories [9]. The particular amount of weight to be gained during pregnancy is subject to the pre-gestational body mass index (BMI) [9]. The guidelines advocate that pregnant women with underweight status should gain a larger amount of weight compared to pregnant women with overweight or obese weight status. However, a reverse phenomenon, where GWG recommendations are being exceeded by pregnant women with overweight or obese weight statuses, has been noted by studies around the world [10,11]. Therefore, identifying patterns of GWG per trimester and overall across the entire pregnancy is crucial for understanding the BMI-segmented trajectory of weight gain or the lack thereof. These findings will enable the development of demographic-specific targeted interventions, supporting more effective weight management [12].

GWG is influenced by an array of factors, including but not limited to dietary intake, physical activity, sleep quality, mental health status, and the presence of pregnancy-related complications [13–15]. Additionally, these associations are complicated by the socio-economic strata divide, where patterns and factors affecting GWG are likely to be different in rural and urban areas [16]. This is due to the disparity in access to healthcare facilities [17], food security [18], and spending behavior concerning populations residing in rural vs urban areas [19]. However, there are limited data on the aforementioned factors and their association with GWG in a prospective longitudinal study design; such a design is required for quantifying the clinical relevance and strength of association.

Currently, IOM guidelines are the only available standards prescribed for appropriate GWG, formulated according to pre-gestational BMI. However, many studies have reported that their respective study population could not follow these guidelines due to their lack of specificity for different ethnicities [20]. It is unclear whether these guidelines apply to countries like India, where the population of pregnant women is predominantly Asian. The applicability is limited because BMI cut-points for Asian individuals are different from those as defined by the World Health Organisation (WHO), based on which IOM guidelines have been formulated. This limits the relevance of the IOM GWG guidelines to Asia-specific populations. Therefore, to address the high neonatal mortality rate in India, there is a critical need to assess the feasibility and utility of IOM guidelines in communities across India.

There is also a scarcity of data on longitudinal patterns and predictors of GWG in a comparative analytical form from a rural vs. urban point of view in India. Maternal nutrition, an important predictor of neonatal health and well-being, is heavily influenced by behavioral and contextual factors, including socioeconomic status and residence [21]. Therefore, it is crucial to understand the maternal nutritional status trajectories for rural and urban pregnant women separately [22]. However, evidence on modifiable individual and contextual factors across different settings is meager, particularly in India. Thus, our objectives were to study the longitudinal patterns and predictors of GWG in pregnant women residing in rural and urban areas in and around Pune city, Maharashtra, India, and to compare them with the pre-existing guidelines provided by IOM, 2009.

## Methods

### Study design and subjects

A longitudinal prospective observational study was carried out in and around Pune city, Maharashtra, Western India. Rural and urban pregnant women (as defined by the Indian state government based on municipalities or corporations, population threshold, and administrative criteria [23,24]) attending randomly selected rural and urban health care centers were screened and enrolled as a part of a community-based cohort called Mother and Infant (*MAI,* which means mother in Marathi- a language native to Maharashtra).

### Sample size calculation

A-priori sample size was calculated to be 300 (150- rural and 150- urban) after accounting for a 20% attrition rate, effect size f^2^ of 0.1 [25], 0.8 power, and alpha 0.05 using a linear multiple regression fixed model (F test family) using the G power software (Version 3.1.9.7). However, after commencing the data collection for the rural arm, an even higher attrition rate was encountered and hence post-hoc sample size re-calculation was performed using the G power software’s linear multiple regression fixed model (F test family) (G power software (Version 3.1.9.7)), with an alpha 0.05 and a small effect size. Ultimately, complete data were obtained on 268 participants (134 rural arm and 134 urban arm) (power of the study remained unchanged at 0.8). This observed attrition emphasizes the challenges of participant retention in rural settings, highlighting the need for deliberation of recruitment and follow-up strategies in future research within similar populations.

### Sample selection

**Fig 1:**
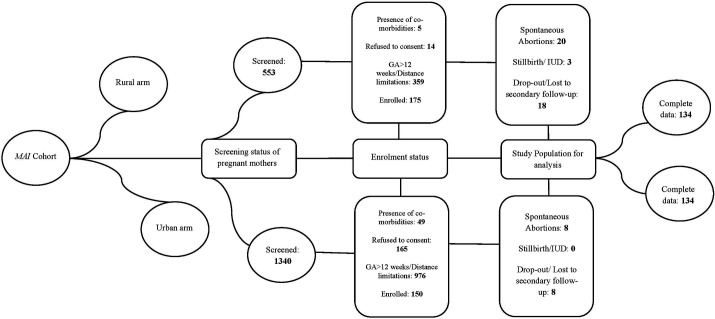
Enrolment flow chart.

Apparently healthy singleton pregnant women, in their first trimester (8–12 weeks of gestation) without the presence of co-morbidities like diabetes mellitus and hypertension, were enrolled in the study. Non-singleton pregnant women having diabetes mellitus or hypertension with a gestational age (calculated using a dating scan) of less than 8 and more than 12 weeks were excluded from the study. The enrolment flowchart of rural and urban pregnant women has been illustrated in Fig 1.

Distance limitation: Pregnant women who planned to give birth in an area more than 150 km away from Pune city were not included in the study due to logistical considerations. Screening to enrollment value was observed to be 5.8 ~ 6, i.e., for enrolling 1 participant, 6 needed to be screened [26].

### Data collection

Data collection was performed between 24^th^ August 2020–26^th^ September 2023. Ethics approval was obtained from the Institutional Ethics Committee before starting data collection (Dated: 02/03/2020, EC approval no.: JCDC/BHR/23/048). Pregnant women were informed about the aim of the study and were provided with the subject information sheet. They were given plenty of opportunities to ask questions before they were enrolled in the study. Written informed consents were obtained from all pregnant women before the initiation of data collection. Data were collected at four time points starting from the first trimester (T1: 8–12 weeks of gestation), the second trimester (T2: 18–22 weeks of gestation), and finally twice during the third trimester (T3_1_: 28–32 weeks of gestation and T3_2_: before parturition when pregnant women were admitted for delivery). All participants were followed up via phone calls and messages at regular intervals to ensure compliance. All the questionnaires were translated into the local regional language of Marathi and a bilingual data collection field researcher administered the questionnaires to the participants using an interview method.

Data on age, location of residence, socio-demography (possession of electricity, two-wheeler/four-wheeler, washing machine, agricultural land, etc.), attributes (physical activity, sleep quality, prenatal distress, diet, clinical data), and anthropometry (height, weight), were obtained once in each trimester.

#### Socio-demography.

Family socioeconomic status (SES) was recorded using the New Socioeconomic Classification system according to the socioeconomic classification, 2011. The system scores families based on the number of material possessions owned, which includes a list ranging from electricity connection to agricultural land, and considers the educational background of the family’s primary earner [27].

#### Anthropometry.

Pregnant women’s weight was recorded using an electronic weighing scale measured to the nearest 0.5 kg, and the measurements were verified with the patient’s medical history. Pre-pregnancy BMI was calculated using self-reported weight from the early first trimester, serving as a proxy for pre-gestational weight. Pregnant women were weighed once in the first and second trimesters and twice during the third trimester. The second measured weight in the third trimester accounted for the final reading to compute the GWG. GWG was calculated by subtracting the weight at baseline from the weight recorded before parturition. Actual weight gain was calculated as the difference between pre-pregnancy weight and the last recorded pregnancy weight. The observed GWG was then compared to the expected range, provided by IOM, 2009 for the same gestational age and BMI category. Based on this, GWG was categorized into inadequate, adequate, and excess weight gain.

Maternal height was recorded to the nearest 0.5 cm using a SECA stadiometer. Furthermore, pre-gestational BMI was calculated using self-reported weight. BMI was computed using the formula: *BMI (kg/m*^*2*^*) = weight (kg)/ height (m)*^*2*^. BMI was computed using recommendations from WHO as follows: underweight weight status: BMI < 18.5 kg/m^2^, normal weight status: BMI 18.5–24.9 kg/m^2^, overweight weight status: BMI 25–29.9 kg/m^2^, and obese weight status: BMI ≥ 30 kg/m^2^ [28]. BMI classification was also performed for comparison based on the WHO cut-points available for Asia-Pacific populations as follows: underweight weight status: BMI < 18.5 kg/m^2^, normal weight status: BMI 18.5–22.9 kg/m^2^, overweight weight status: BMI 23–24.9 kg/m^2^, and obese weight status: BMI ≥ 25 kg/m^2^ I [29]. Classification using South Asian BMI cut-points was also included as follows: underweight weight status: BMI < 18.5 kg/m^2^, normal weight status: BMI 18.5–23 kg/m^2^, overweight weight status: BMI 23–27.5 kg/m^2^, and obese weight status: BMI ≥ 27.5 kg/m^2^.

The Institute of Medicine provided guidelines for GWG based on pre-gestational BMI in 2009 [28]. The recommended weight gain for pregnant women based on weight status is 12.5−18 kg for underweight, 11.5−16 kg for normal, 7–11.5 kg for overweight, and 5−9 kg for obese women. Similarly, a trimester-specific weight gain range is provided according to pre-gestational BMI by the IOM. Mean weight gain (kg) (range (kg)) for pregnant women based on weight status has been suggested as follows: 0.51 (0.44–0.58) for underweight, 0.42 (0.35–0.50) for normal, 0.28 (0.23–0.33) for overweight and 0.22 (0.17–0.27) for obese weight status [28]. To ensure accuracy, BMI classification from the WHO, Asia-Pacific, and South Asian cut-offs was applied to the Indian pregnant women in our study. This approach allowed us to categorize weight gain as inadequate, adequate, or excessive based on comparisons with the IOM’s recommended weight gain targets for each trimester.

#### Physical activity.

Data on physical activity were recorded once in each trimester using a standardised semi-quantitative questionnaire, the pregnancy-specific physical activity questionnaire (PPAQ), which has been validated for use in the Indian population [30,31]. This is a self-administered questionnaire that asks the respondents to report time spent in a variety of 32 activities per week, including sports/exercise, inactivity, household/caregiving, transportation, and occupation during the current trimester. Pregnant women were asked to select the duration spent in each activity, which ranged from 0 to 3 or more than 3 hours. The Metabolic Equivalent of Task (MET) in hours/week was derived based on the duration and frequency of each activity that was being engaged in. METs attributed to the intensity of activity (sedentary, light, moderate, and vigorous activity) and type of activity (household, transport, occupation, and exercise-related) were calculated and used for the final analysis. For scoring, we followed the standard PPAQ scoring protocol. Predefined duration scores (e.g., 0.25, 0.75, 1.5, 2.5, 3.0) were assigned to each activity as per the original scoring guidelines. These values were then multiplied by the corresponding MET intensity scores to calculate average weekly energy expenditure (MET-hours/week). The MET values corresponded to the intensity of each activity (sedentary, light, moderate, or vigorous) and the type of activity (household, transport, occupational, and exercise-related) and were used for the final analysis. Further, an additional validated 24-hour physical activity questionnaire was also used, which measured hours attributed to sleep, screen time, and minutes spent in leisure time, along with minutes spent engaging in sedentary, light, moderate, and vigorous activity.

#### Sleep quality.

Sleep quality was recorded once in each trimester using a standardized questionnaire: the Pittsburgh Sleep Quality Index, a reliable tool for measuring subjective sleep quality, which has been used in the Indian population [32–34]. It measures different domains of sleep, namely sleep latency, subjective sleep quality, habitual sleep efficiency, sleep duration, sleep disturbance, daytime dysfunction due to sleepiness, and the use of medication over the past month. Each parameter of sleep quality is a score, ranging from 0 to 3. Based on these domains, sleep was categorized as either good or poor (a combined score of more than or equal to 5 is poor sleep, and less than 5 is good sleep).

#### Prenatal distress.

Prenatal distress was measured using a validated: the prenatal distress questionnaire [35]. It is a 3-point Likert scale that takes concerns regarding pregnancy into account, which are categorized into several domains: concerns about the baby, childbirth, physical and social changes during pregnancy, finances, healthcare quality, health status, care of the baby, and post-partum life. The scale has a range of 1 = no distress to 3 = high distress, where the global score ranges from 17 to 51 (the higher the score, the higher the distress). Since PDQ, to the best of our knowledge, has not been previously used in Indian populations, we assessed the reliability of the language-translated PDQ in a sample of 20 pregnant women from rural and urban areas and found Cronbach’s alpha to be 0.8.

#### Dietary data.

Data on dietary intake were collected by a 3-day (2 non-consecutive weekdays and 1 weekend day) dietary recall, using a multiple pass method administered by a trained dietitian acquainted with local foods. Food quantity was estimated using standard cups, plates, and spoons. Nutrient intakes were calculated using a raw and cooked food database software C-Diet (version 3.0) [36]. Recorded dietary macronutrient data were compared with reference guidelines provided by the Indian Council of Medical Research, National Institute of Nutrition, Hyderabad, India [37].

### Statistical analysis

Statistical analysis was performed using the Statistical Package for Social Sciences (IBM SPSS), version 27. Normality tests were conducted on all variables using the Kolmogorov-Smirnov test before performing analysis. We handled the missing data by replacing the missing values with the mean of that column [38]. Normal continuous variables were expressed as mean (+/- standard deviation (S.D.)), and categorical variables were expressed as percentages. T-test was used to assess the significance of the difference between the means of variables and for assessing the patterns of GWG across rural and urban locations. Pearson’s correlation coefficient was used to express the correlation between continuous variables. We categorized the rural and urban populations as gaining inadequate or excess weight in the second and third trimesters based on IOM cut-points. Logistic regression analysis was run to predict the trimester-specific predictors of inadequate and excess weight gain. The level of significance was set at a *p-*value of < 0.05.

## Results

Data on 268 pregnant women were analyzed (134 rural and 134 urban), with mean maternal age being 23.0 ± 5.8yrs. Rural pregnant women married young, conceived early, were shorter, and had lower body weights compared to urban pregnant women. Moreover, urban pregnant women were found to be more educated compared to their rural counterparts (*p* < 0.05). 4.5% of rural pregnant women were reported to be illiterate. 16% of rural pregnant women and 56% of urban pregnant women worked in a job, while 84% of rural pregnant women and 44% of urban pregnant women were homemakers (p < 0.05). 52.2% rural pregnant women were primigravida, and 47.8% were multigravida, and similarly, an almost equal proportion of urban pregnant women were primigravida (48.5%) as well as multigravida (51.5%). Urban and rural differences in the study population are illustrated in Table 1a.

**Table 1:** a. Urban and rural characteristics in demographic parameters of pregnant women in the MAI Cohort. b. Urban and rural differences in physical activity, sleep quality, prenatal distress, and dietary intake of pregnant women across trimesters in MAI Cohort.

Parameter	Rural (n = 134)		Urban (n = 134)		Total (n = 268)	*p*-value
Maternal age at pregnancy (years)	23.4 ± 3.7		30.9 ± 3.8		27.1 ± 5.3	0.01
Maternal education n (%)						
Less than 15 years of formal education	124 (70.9)		10 (6.7)		134 (41.2)	<0.05
At least 15 years of formal education and above	51 (29.1)		140 (93.3)		191 (58.8)	<0.05
Socio economic status n (%)						
Low socio-economic status	43 (32.1)		–		43 (16)	<0.05
High socio-economic status	91 (67.9)		118 (100)		225 (84)	<0.05
Parity						
0	79 (59)		112 (83.6)		191 (71.3)	<0.05
1	50 (37.3)		20 (14.9)		70 (26.1)	<0.05
≥2	5 (3.7)		2 (1.5)		7 (2.6)	>0.05
Maternal pregnancy weight (kg)	48.6 ± 10.1		61.8 ± 11.7		55.3 ± 12.6	0.01
Maternal height (cm)	153.6 ± 5.6		155.9 ± 5.3		154.8 ± 5.5	0.01
Maternal pre-gestational BMI (kg/m2)	20.5 ± 4.0		25.5 ± 4.7		23 ± 5.0	0.01
Maternal pre-gestational BMI n (%) (WHO cut-points)						
Underweight BMI	49 (36.6)		10 (8.5)		59 (22)	<0.05
Normal BMI	68 (50.7)		45 (38.1)		117 (43.7)	<0.05
Overweight BMI	12 (9)		45 (38.1)		68 (25.4)	<0.05
Obese BMI	5 (3.7)		18 (15.3)		24 (9.0)	<0.05
Maternal pre-gestational BMI n (%) (Asian Pacific cut-points)						
Underweight BMI	49 (36.6)		10 (7.5)		59 (22)	<0.05
Normal BMI	57 (42.5)		29 (21.6)		86 (32.1)	<0.05
Overweight BMI	11 (8.2)		23 (17.2)		34 (12.7)	<0.05
Obese BMI	17 (12.7)		72 (53.7)		89 (33.2)	<0.05
Maternal pre-gestational BMI n (%) (South Asian cut-points)						
Underweight BMI	49 (36.6)		10 (7.5)		59 (22)	<0.05
Normal BMI	57 (42.5)		29 (21.6)		86 (32.1)	<0.05
Overweight BMI	18 (13.4)		55 (41)		73 (27.2)	<0.05
Obese BMI	10 (7.5)		40 (29.9)		80 (18.7)	<0.05
**Parameter**	**1**^**st**^ **trimester**		**2**^**nd**^ **trimester**		**3**^**rd**^ **trimester**	
	**Rural**	**Urban**	**Rural**	**Urban**	**Rural**	**Urban**
Sedentary physical activity (MET Hrs/week)	5.7 ± .7^*^	16.2 ± 5.8	6.1 ± 4.0^*^	16.2 ± 5.5	6.6 ± 3.8^*^	15.5 ± 4.7
Light physical activity (MET Hrs/week)	15.5 ± 8.8^*^	10.5 ± 7.0	16.1 ± 7.9^*^	10.2 ± 6.4	14.9 ± 7.2^*^	9.6 ± 5.7
Moderate physical activity (MET Hrs/week)	9.6 ± 7.6	9.4 ± 11.2	9.5 ± 8.8	9.2 ± 8.9	9.9 ± 7.4	8.8 ± 7.4
Vigorous physical activity (MET Hrs/week)	0.5 ± 1.5^*^	0.0 ± 0.0	0.4 ± 1.5^*^	0.0 ± 0.3	0.2 ± 0.8	0.2 ± 1.6
Household/ caregiving- related activity (MET Hrs/week)	20.0 ± 13.0^*^	16.1 ± 16.0	19.8 ± 11.0^*^	13.9 ± 11.4	20.3 ± 10.3^*^	13.2 ± 9.5
Occupational/job- related activity (MET Hrs/week)	1.2 ± 4.1^*^	7.5 ± 9.3	1.2 ± 4.8^*^	8.8 ± 10.9	0.1 ± 1.4^*^	6.7 ± 10.3
Sports/exercise- related activity (MET Hrs/week)	4.2 ± 3.8^*^	1.0 ± 1.5	4.5 ± 3.6^*^	1.2 ± 1.1	4.0 ± 3.5^*^	1.8 ± 2.4
Global Pittsburgh Sleep Quality Index score	5.3 ± 3.3^*^	4.4 ± 3.6	4.7 ± 3.0^*^	3.8 ± 3.3	5.8 ± 3.7	5.0 ± 2.9
Prenatal distress score	31.4 ± 7.8^*^	29.0 ± 10.5	31.8 ± 8.6^*^	26.3 ± 10.3	32.7 ± 8.5	32.9 ± 10.4
Energy (kcal)	1337 ± 380^*^	1638 ± 368	1738 ± 365^*^	2064 ± 412	2003 ± 432^*^	2379 ± 398
Protein (g)	30 ± 10^*^	38 ± 10	36 ± 10^*^	47 ± 13	39 ± 12^*^	49 ± 12
Carbohydrate (g)	202 ± 56^*^	245 ± 58	239 ± 58^*^	277 ± 61	251 ± 65^*^	295 ± 59
Fat (g)	36 ± 18^*^	47 ± 15	43 ± 13^*^	57 ± 18	47 ± 17^*^	62 ± 17

BMI: Body Mass Index, GWG: Gestational Weight Gain, WHO: World Health Organization.

Data expressed as Mean±SD.

* Differences were statistically significant across rural and urban settings per trimester and overall across the entire pregnancy at *p* < 0.05.

### Characteristics of physical activity, dietary intake, sleep quality, and prenatal distress of rural and urban pregnant women

Urban pregnant women reported a higher screen time (time spent on mobile, television, laptop, etc.) (236 minutes/ day) (median (IQR: 210 (150))) compared to rural pregnant women (122 minutes/ day) (median (IQR: 120 (74))) (p value=<0.05). Trends in physical activity, sleep quality, prenatal distress, and dietary intakes of rural and urban pregnant women have been described in Table 1b. Higher levels of sedentary activity were observed among urban pregnant women, while rural pregnant women were more frequently engaged in household/caregiving activities (*p* < 0.05).

Urban pregnant women reported significantly better sleep quality than their rural counterparts (*p* < 0.05). Rural and urban pregnant women reported an average of 7 hours of sleep throughout gestation. 80.5% of all pregnant women reported sleep disturbances in the first trimester, which later increased over gestation (second trimester: 83% and third trimester: 95%). Nearly 42% of all pregnant women reported high prenatal distress during pregnancy, with rural pregnant women having higher distress compared to their urban counterparts (51% vs. 33%, *p*-value <0.05). The highest distress was observed in the third trimester (52% vs 48%) across both groups (*p* > 0.05).

Dietary intakes were higher in urban pregnant women compared to rural counterparts (*p* < 0.05) (Table 1b). When comparing the dietary intakes with reference values (National Institute of Nutrition, Indian Council of Medical Research), significant differences in forms of dietary energy, protein, carbohydrate, and fat intakes were noted across all trimesters (*p* < 0.05). When comparisons were made to location, it was observed that rural pregnant women complied with dietary energy recommendations (estimated average requirement: 2010 kcal for all trimesters) in the third trimester, whereas urban pregnant women complied in the second trimester. (*p* > 0.05). Upon further analysis, it was observed that, overall, everyone consumed inadequate protein (estimated average requirement: 36 g in the first trimester, 44 g in the second trimester, and 54 g in the third trimester) throughout pregnancy, with the majority being rural pregnant women. Dietary carbohydrate consumption (estimated average requirement: minimum 130 g for all trimesters) and fat (estimated average requirement: 30 g visible fat for all trimesters) were largely sufficient (*p* > 0.05).

### Patterns of GWG among rural and urban pregnant women

The general WHO BMI guidelines, along with WHO BMI guidelines using Asia-Pacific and South-Asian cut-points, were compared against the recommended guidelines for GWG. It was observed that the rate of weight gain was the highest in the second trimester (*p* < 0.05). Weight gain was higher in urban pregnant women compared to rural counterparts (*p *< 0.05). 61.2% rural underweight pregnant women gained inadequate weight, whereas only 30% urban underweight weight status pregnant women gained inadequate weight (*p* < 0.05). Only 11.8% of overweight or obese pregnant women in the rural arm exceeded recommended GWG guidelines, compared to 57.3% of pregnant women in the urban arm (*p *< 0.05). Thus, as per the IOM guidelines, 44% of pregnant women gained inadequate weight, 33.2% gained adequate weight, and 22.8% exceeded recommended GWG guidelines (Table 2). Furthermore, GWG based on the Asia Pacific and South Asian BMI classification has also been illustrated in Table 2.

**Table 2:** Patterns of GWG of urban and rural pregnant women in MAI Cohort.

	Rural (n = 134)	Urban (n = 134)	Total (n = 268)
Weight gain at 1^st^ trimester (kg)	2.0 ± 1.8*	3.0 ± 2.4	2.5 ± 2.2
Weight gain at 2^nd^ trimester (kg)	4.4 ± 2.1	4.4 ± 2.2	4.4 ± 2.1
Weight gain at 3rd trimester (kg)	3.4 ± 1.7*	4.4 ± 2.1	3.9 ± 2.0
Maternal total GWG (kg)	9.9 ± 3.7*	11.9 ± 4.5	10.9 ± 4.2
GWG Categories n (%) (WHO BMI classification)			
Inadequate GWG	80 (59.7) *	38 (28.4)	118 (44)
Adequate GWG	45 (33.6)	44 (32.8)	89 (33.2)
Excess GWG	9 (6.7)*	52 (38.8)	61 (22.8)
GWG Categories n (%) (Asia-Pacific BMI classification)			
Inadequate GWG	75 (56)	25 (18.7)	100 (37.3)
Adequate GWG	42 (31.3)	40 (29.9)	82 (30.6)
Excess GWG	17 (12.7)	69 (51.5)	86 (32.1)
GWG Categories n (%) (South-Asian BMI classification)			
Inadequate GWG	76 (56.7)	27 (20.1)	103 (38.4)
Adequate GWG	45 (33.6)	43 (32.1)	88 (32.8)
Excess GWG	13 (9.7)	64 (47.8)	77 (28.7)

* **Significantly different from urban counterparts at *p*-value of <0.05.**

We observed that overall, pregnant women in our cohort gained a mean (S.D. of 10.9) 4.2 kg with a range of −2.9 kg −25.5 kg. Pregnant women whose pre-pregnancy BMI categorized their weight status as underweight gained 11.1 kg (S.D. of 3.7) with a range of 4.8 kg-25.5 kg, normal weight status pregnant women gained 10.8 kg (S.D. of 4.0) with range of 1.1 kg-19.7 kg, overweight weight status pregnant women gained 11.6 kg (S.D. of 4.7) with a range of −1.1 kg-24.6 kg and obese weight status pregnant women gained 8.6 kg (S.D of 4.6) with a range of −2.9 kg −18.9 kg. Rural pregnant women with underweight (4.8 kg-19.8 kg vs 5.4 kg-25.5 kg) and normal (1.1 kg-17.9 kg vs. 5.3 kg −19.7 kg) weight statuses gained less weight in comparison to their urban counterparts (*p* < 0.05). Urban overweight and obese pregnant women gained weight within a range of −1.1 kg-24.6 kg and −2.9 kg-18.9 kg, respectively, whereas their rural counterparts gained weight within a range of 3.6 kg- 13.6 kg and 3.4 kg- 8.9 kg, respectively (*p* < 0.05).

### Comparison of GWG of rural and urban pregnant women with IOM guidelines

**Fig 2:**
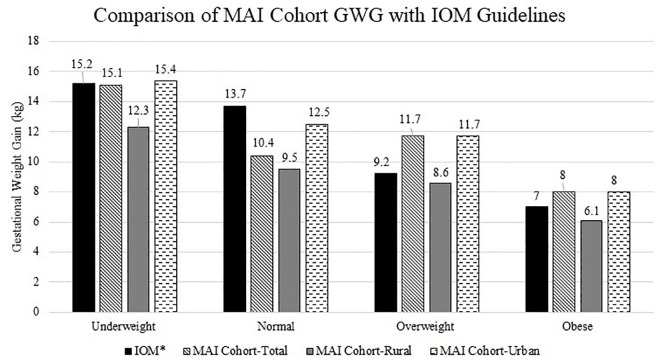
Comparison of MAI Cohort GWG with IOM guidelines.

Overall, it was observed that pregnant women of underweight and normal weight status gained insufficient weight as per IOM guidelines and that overweight and obese pregnant women exceeded recommended GWG guidelines as per IOM guidelines (*p* < 0.05) (Fig 2). Upon further analysis, we found that rural pregnant women across all pre-gestational BMI categories gained less weight than recommended by IOM guidelines (*p* < 0.05). Conversely, urban pregnant women of underweight and overweight/obese pre-gestational weight statuses exceeded these guidelines, while those with a normal weight status gained less than recommended(*p* < 0.05).

### Relationship of GWG with lifestyle factors

We examined the relationship between GWG and lifestyle factors such as physical activity, dietary intake, prenatal distress, and sleep quality (Table 3).

**Table 3:** Correlation of GWG with trimester-specific parameters of pregnant women in the MAI Cohort.

Parameter	Trimester I (r)	Trimester II (r)	Trimester III (r)
Maternal height	0.180^**^	–	–
Sedentary physical activity	0.129^*^	0.237^**^	0.196^**^
Light-moderate physical activity	−0.187^**^	−0.125^*^	NS
Household/caregiving activity	−0.179^**^	−0.126^*^	NS
Occupational activity	NS	0.144^*^	NS
Sports/exercise activity	NS	−0.226^**^	NS
Dietary energy	0.142^*^	0.305^*^	0.317^*^
Dietary protein	0.122^*^	0.255^*^	0.235^**^
Dietary carbohydrates	0.131^*^	0.252^*^	0.224^*^
Dietary fat	0.208^*^	0.238^*^	NS
Prenatal distress	NS	−0.122^*^	NS
Sleep quality	NS	NS	NS

NS: Not Significant.

* Correlations significant at *p* value of <0.05.

** Correlations significant at *p* value of <0.001.

### Predictors of GWG

An interaction term between BMI distribution and location was included in the logistic regression model to assess effect modification. The interaction was statistically significant (B = −0.279, p = 0.001), indicating that the association between BMI distribution and gestational weight gain differs by rural vs. urban residence. Thus, since location of residence of pregnant women was observed to be an important predictor of GWG (*p* < 0.05); the models were run separately for pregnant women residing in rural and urban areas (Table 4). We conducted two separate binary logistic regression analyses with inadequate and excess GWG (yes/no) as outcomes, defined based on BMI-specific IOM 2009 guidelines. Prepregnancy BMI was included both for outcome classification and as a predictor.

**Table 4:** Predictors of inadequate and excess weight change of pregnant women in MAI Cohort.

Parameter	B	OR	95% CI		*p* value	B	OR	95% CI		*p* value
			Lower	Upper				Lower	Upper	
**Inadequate weight change at 2**^**nd**^ **trimester**										
	**Rural**					**Urban**				
Maternal age	0.01	1.01	0.90	1.14	0.83	−0.02	0.97	0.87	1.08	0.61
Socio economic status										
Low	0.84	2.33	0.97	5.59	**0.05**	.	.	.	.	.
High	Reference									
Parity	1.07	2.93	1.26	6.78	**0.01**	−0.09	0.91	0.34	2.39	0.85
Pre-pregnancy BMI (Kg/m^2^)										
Underweight	1.69	5.43	2.13	13.84	**0.0001**	−0.09	0.90	0.21	3.90	0.89
Overweight/ Obese	−1.39	0.24	0.06	1.01	0.05	−0.95	0.38	0.16	0.89	**0.02**
Normal	Reference									
Dietary protein										
Inadequate	0.91	2.48	1.12	6.38	0.06	0.24	1.28	0.58	2.81	0.53
Adequate	Reference									
Sedentary physical activity	−0.01	0.99	0.97	1.00	0.25	−0.00	0.99	0.98	1.01	0.72
Prenatal distress	−0.02	0.97	0.93	1.02	0.31	0.03	1.03	0.99	1.07	0.10
Sleep quality										
Poor sleep	0.98	2.68	1.12	6.38	**0.02**	0.10	1.11	0.48	2.58	0.80
Good sleep	Reference									
**Excess weight change at 2**^**nd**^ **trimester**										
Maternal age	0.008	1.00	0.87	1.16	0.91	0.01	1.01	0.91	1.12	0.78
Socio economic status										
Low	−0.75	0.46	0.15	1.44	0.18	.	.	.	.	.
High	Reference									
Parity	−0.76	0.46	0.16	1.31	0.14	−0.44	0.63	0.23	1.75	0.38
Pre-pregnancy BMI (Kg/m^2^)										
Underweight	−0.74	0.47	0.15	1.42	0.18	0.37	1.44	0.24	8.52	0.68
Overweight/ Obese	2.35	10.53	2.37	46.75	**0.002**	2.00	7.40	2.96	18.51	**0.0001**
Normal	Reference									
Dietary protein										
Inadequate	−0.63	0.53	0.17	1.60	0.26	0.07	1.07	0.49	2.36	0.85
Adequate	Reference									
Sedentary physical activity	0.01	1.01	0.99	1.03	0.18	0.002	1.00	0.99	1.01	0.74
Prenatal distress	0.05	1.05	0.99	1.11	0.08	−0.01	0.99	0.95	1.02	0.59
Sleep quality										
Poor sleep	−1.75	0.17	0.05	0.55	**0.003**	−0.22	0.79	0.34	1.85	0.74
Good sleep	Reference									

The models were adjusted for maternal age, socio-economic status and parity, OR: Odd’s ratio for the likehood of inadequate and/or excess weight gain.

We observed that significant predictors of inadequate weight change in the 3rd trimester were parity (OR: 2.85, 95% CI: 1.2–6.7) and poor sleep (OR: 3.30, 95% CI: 1.26–8.62) (*p* < 0.05). Furthermore, significant predictors of excess weight change in the third trimester were parity (OR: 0.21, 95% CI: 0.06–0.73) and overweight or obese pre-gestational BMI (OR: 7.61, 95% CI: 3.02–19.18) (*p* < 0.001). These results were consistent across rural and urban locations. Moreover, the effect was more pronounced using Asian and South Asian BMI cut-points, as previously displayed in Table 2.

## Discussion

GWG is an important predictor of intrauterine fetal and neonatal growth [3]. Factors influencing GWG largely vary across different populations and locations worldwide [39]. Therefore, identifying the specific factors that determine adequate gestational gains for pregnant women in India is a crucial step in combating inadequate/excess GWG-related adverse pregnancy outcomes. Our longitudinal study bridges this gap in research and explores trimester-wise socio-economically bifurcated predictors of GWG. We have observed that multiparous rural pregnant women with underweight weight status who experience poor sleep were more prone to inadequate weight gain during pregnancy compared to their urban counterparts. We found that nulliparous urban pregnant women with overweight or obese weight status were more likely to gain excess weight during pregnancy. Rural pregnant women were less likely to gain weight within IOM recommendations irrespective of their pre-gestational weight status, whereas urban pregnant women were more likely to meet recommendations.

In our cohort, we found that rural pregnant women gained less weight compared to their urban counterparts and that the majority of underweight pregnant women gained inadequate weight according to IOM guidelines. Also, urban pregnant women were at a higher risk of excessive GWG. Similar findings were reported in the study done by Dai *et al.*; that is, compared to rural pregnant women, urban pregnant women had higher odds of experiencing excessive GWG [40]. This diverges from the findings from studies by Tavernier *et* al [41] and Gallagher *et al* [42], who report that rural pregnant women had a higher chance of being overweight or obese. This variability may be attributed to the fact that most pregnant women in the study by Gallagher *et al.*’s study resided in rural areas and were exposed to an unhealthy lifestyle. Conversely, our rural population was found to engage in an active and healthy lifestyle, which was protective against excessive GWG.

We observed that both rural and urban underweight pregnant women in our cohort experienced the highest GWG, followed by other BMI categories. Similar findings were reported in studies by Rodrigues *et al*. regarding underweight pregnant women experiencing the highest GWG followed by normal and finally overweight and obese pregnant women [43]. Interestingly, we also observed that while underweight and normal weight status pregnant women experienced higher GWG than pregnant women in other categories, irrespective of location, they still gained inadequate weight when compared with the IOM guidelines.

Upon comparing GWG as per location, we observed that rural pregnant women, irrespective of pre-gestational weight status, gained inadequate weight compared to IOM guidelines. In the study conducted by Daemers *et al.* [44], it was reported that normal-weight-status pregnant women were at a higher risk of gaining less than the recommended weight, while obese weight-status pregnant women were at the risk of gaining both inadequate as well as excess weight. However, their study population consisted of clinically healthy, low-risk pregnant women from the USA and Netherlands, who thus likely had varying risk profiles and lifestyles. This demographic diversity could have created a bias in the distribution of pre-gestational BMI, as a mere 1.4% of the study population experienced an underweight weight status. We observed that urban pregnant women of underweight, overweight, and obese pre-gestational weight status exceeded recommended GWG guidelines as per IOM guidelines, while those with normal pre-gestational weight status gained less weight than outlined by the IOM guidelines. This contrasts with the findings of the study conducted by Arora et al. [45]. The discrepancy may be partly explained by our study being conducted during the COVID-19 pandemic; previous literature suggests that pregnant women gained excessive gestational weight during the period of COVID-19 [46,47].

In the study by Arora et al. on pregnant women belonging to urban areas, close to half of the participants (48.12%) exceeded recommended GWG guidelines as per IOM guidelines. However, their BMI classification was based on WHO Asian-Indian cut-points, which differ from the general WHO cut-points, which may have led to an overestimation of the proportion of pregnant women who exceeded recommended GWG guidelines compared to our study. Nevertheless, their methodology provides a more relevant criterion for evaluating the study population. Furthermore, Arora *et al*. ’s study population consisted of pregnant women belonging to upper socio-economic status, who may have had greater access to processed foods and ready-to-eat meals in comparison to individuals with poor socio-economic status. This increased access may explain the observed excess GWG among study participants. In our cohort, we observed that when pre-pregnancy BMI was categorized based on the Asia-Pacific guidelines and South Asian guidelines, respectively, among rural and urban pregnant women, the percentage prevalence of pre-gestational overweight and obesity was higher compared to the results derived from WHO BMI cut-points. This effect was more pronounced in urban pregnant women, largely in those who exceeded recommended GWG guidelines according to the IOM guidelines. This highlights that when using WHO BMI guidelines, the biological predictors of Asian individuals’ health statuses, such as being more genetically predisposed to adiposity, are overlooked [48,49]. Since we found that pre-gestational BMI was a significant predictor of GWG, our study suggests that there may be a need for ethnic-specific normative data. Although our findings are specific to rural and urban Indian pregnant women, it is important to acknowledge the broader debate surrounding race- and ethnicity-based clinical recommendations. Similar to ongoing discussions about BMI thresholds for Asian populations, calls for ethnicity-specific GWG guidelines must be approached with caution. Any such recommendations should account for the complex interplay of sociocultural, environmental, nutritional, and healthcare access factors, rather than attributing GWG differences solely to ethnicity.

Physical activity is crucial for maintaining a healthy pregnancy weight and preventing the chances of cesarean section, gestational diabetes mellitus, depressive disorders during pregnancy, operative vaginal deliveries, etc. [50]. We observed a moderate positive association between sedentary activity and GWG; however, the effect was not strong enough to be statistically significant in the GWG prediction model. This may be attributed to the small sample size in the current cohort, which was selected to achieve statistically significant results. However, various studies on the relationship between physical activity and GWG have shown mixed and inconsistent results. Ruifrok *et al*. reported no association between sedentary activity and GWG [51], while Stuebe *et al.* found that mid-pregnancy walking and vigorous physical activity were inversely associated with excessive GWG [52].

We also observed that urban and rural pregnant women consuming insufficient dietary protein were twice as likely to experience inadequate weight gain. Likewise, studies have reported that diet quality plays an important role in gaining appropriate weight by reducing the odds of inadequate or excess weight [53,54]. Our findings suggest that dietary protein plays a protective role in preventing excess GWG, a finding supported by a study conducted by Rugina *et al.,* which demonstrated the importance of a protein-based diet [55]. Additionally, a study by Shin *et al.* reported that reduced dietary protein intake was associated with a higher risk of inadequate GWG [56].

The impact of sleep quality on GWG is an area that is less explored, especially in India. We found that poor sleep was a significant contributor to an increased odds of inadequate weight gain in rural and urban pregnant women. Similarly, a study conducted by Hawkins *et al.,* which aimed to assess the association of multidimensional sleep health and GWG, reported that poor individual indicators of sleep were the strongest predictors of low GWG in nulliparous women [57]. Conversely, a study conducted by Hill *et al.* did not find any association between sleep quality and GWG adequacy. However, they reported a positive relationship between longer sleep durations during late pregnancy and the odds of inadequate GWG [58], a finding similar to ours. Moreover, we found that prenatal distress was negatively associated with GWG; however, it was not a significant predictor for GWG, with an inverse relationship between prenatal distress and GWG being observed in the second trimester. In the study conducted by Kominiarek *et al.,* similar findings of low prenatal distress being associated with adequate GWG were reported [59]. This could be attributed to the fact that stress leads to the activation of the hypothalamic-pituitary-adrenal axis, leading to increased glucocorticoid levels, thus increasing adiposity, thereby being reflected in excessive GWG [60].

In our cohort, we observed that parity played a significant role in increasing the odds of inadequate weight gain in the second trimester for rural pregnant women and in the third trimester for urban pregnant women. Similar findings were reported by Paulino *et al.*, Chiba *et al.,* and Cheng *et al.* [61] where mean GWG was higher in primiparous women compared to multiparous women [61–63]. This may be attributed to primigravida’s low level of knowledge and use of unreliable sources for nutritional information, thus resulting in poor dietary choices. While they may intend to be cautious concerning antenatal care, due to the lack of guidance from healthcare professionals, they may consume high-risk foods, demonstrate a lack of desire to make changes to their current dietary choices, and remain unaware of what constitutes healthy GWG [64]. Socio-economic status was found to play a crucial role in modulating GWG in our cohort, and similar findings were reported by Ouédraogo *et al.* [65] and Cheney *et al.* [66].

To the best of our knowledge, this is the only longitudinal study conducted in the global south to examine trimester-wise predictors of inadequate and excess weight gain in rural and urban Indian pregnant women residing in and around Pune, Maharashtra, India, using WHO, Asia Pacific, and South Asian BMI categorizations. Given the limitations of the IOM guidelines in accurately classifying BMI for this population, these alternative categorizations provide a more appropriate framework for assessing GWG. These findings have the potential to inform public health policies for promoting optimal weight gain in diverse rural and urban populations globally. We adopted a holistic approach while predicting factors affecting GWG by examining the effects of various characteristics on pregnancy physiology in rural and urban pregnant women. Our analysis accounted for various characteristics, including dietary intake, physical activity, psychological stress, sleep quality, socio-demographic factors, anthropometric measurements, and clinical and reproductive history. We have highlighted the importance of optimal dietary intake, appropriate physical activity, good-quality sleep, and a healthy state of mind for gaining adequate weight during pregnancy. These factors are critical for maternal and fetal health outcomes and can guide clinical practices by promoting individualized care plans that cater to the unique needs of pregnant women. Additionally, they may provide insights for evidence-based policy formulation to improve maternal health outcomes. We have used standardized and validated tools and instruments for collecting data. Our longitudinal study design can establish cause-effect relationships critical for translating findings to clinical practice. Although a larger sample size would have enhanced the generalizability of the results, our study was adequately powered to generalize findings to the entire population. We studied urban and rural pregnant women separately, highlighting that those residing in rural areas are not meeting IOM recommendations, a finding that is critical when clinically treating populations from environmentally diverse regions of India.

Our study is not without its limitations; our study attrition rates were high, and hence, sample size re-calculation needed to be performed; however, the power of the study remained unchanged, ensuring that the results were unaffected. We considered early first-trimester weight as baseline weight, which may have introduced recall bias in the measurement of GWG as it was self-reported, however, we assessed measurement accuracy by cross-checking our data with participants’ medical histories. Additionally, studies [67] report that very little weight is gained in the first trimester, and therefore, the measurement is often considered a proxy for pre-gestational weight. One limitation of our physical activity assessment method is the use of midpoint values for duration categories. While this approach is consistent with earlier applications of the PPAQ, more recent recommendations suggest using the lower bound of each range to minimize bias from potential over-reporting. This is especially relevant for individuals with low levels of physical activity, where the risk of overestimation may be greater. Future studies may consider applying this modification for improved accuracy. We have used PDQ for measuring prenatal distress of pregnant women, and while the PDQ has not yet been validated for the Indian population, it has been widely used in studies involving pregnant populations. Future research should focus on evaluating its validity within the Indian context. Future research should focus on investigating the impact of the predictors of GWG on maternal-neonatal outcomes. To obtain more actionable insights, future studies could measure more specific and dynamic measures of pregnancy-induced physiological changes, such as the predictors of changes in body composition, enabling an in-depth analysis of pregnancy biology.

### Conclusion

We have highlighted varying trends of GWG for rural and urban pregnant women per trimester and overall across the entire pregnancy and at an overall level. Our findings indicate that urban underweight pregnant women experienced the highest weight gain per trimester and overall across pregnancy and total GWG compared to other BMI-categorized groups. We have identified predictors of GWG that are important modifiable factors, such as sleep, parity, and pre-gestational BMI, which facilitate adequate and optimal gestational weight gain in the second and third trimesters. Given that our findings are based on a single geographical location and timeframe, further research is warranted to better understand how contextual factors such as location of residence may influence GWG patterns and whether tailoring recommendations to such factors could enhance maternal health strategies.

## Supporting information

S1 File STROBE checklist. (DOCX)

S2 File Inclusivity-in-global-research-questionnaire. (DOCX)

## References

[1] Soma-Pillay P, Nelson-Piercy C, Tolppanen H, Mebazaa A. Physiological changes in pregnancy. Cardiovasc J Afr. 2016;27(2):89–94. doi: 10.5830/CVJA-2016-021 27213856 PMC4928162

[2] Ministry of Health and Family Welfare Government of India. National family health survey (NFHS-5). 2019. https://main.mohfw.gov.in/sites/default/files/NFHS-5_Phase-II_0.pdf

[3] Deshpande M, D M, Shah N, Kajale N, Angom J, Bhawra J. Influence of parental anthropometry and gestational weight gain on intrauterine growth and neonatal outcomes: Findings from the MAI cohort study in rural India. PLOS Glob Public Health. 2023;3(8):e0001858. doi: 10.1371/journal.pgph.0001858 PMC10461821 37639449

[4] Bauserman MS, Bann CM, Hambidge KM, Garces AL, Figueroa L, Westcott JL, et al. Gestational weight gain in 4 low- and middle-income countries and associations with birth outcomes: a secondary analysis of the Women First Trial. Am J Clin Nutr. 2021;114(2):804–12. doi: 10.1093/ajcn/nqab086 33876178 PMC8326045

[5] Deierlein AL, Messito MJ, Katzow M, Berube LT, Dolin CD, Gross RS. Total and trimester-specific gestational weight gain and infant anthropometric outcomes at birth and 6 months in low-income Hispanic families. Pediatr Obes. 2020;15(3):e12589. 10.1111/ijpo.12589 PMC7012708 31696650

[6] Surita FG de C, Souza RT, Carrilho TRB, Hsu L de PR, Mattar R, Kac G. Guidelines on how to monitor gestational weight gain during antenatal care. Rev Bras Ginecol Obstet. 2023;45(2):104–8. doi: 10.1055/s-0043-1766109 36977408 PMC10078888

[7] Deputy NP, Sharma AJ, Kim SY, Hinkle SN. Prevalence and characteristics associated with gestational weight gain adequacy. Obstet Gynecol. 2015;125(4):773–81. doi: 10.1097/AOG.0000000000000739 25751216 PMC4425284

[8] Kominiarek MA, Peaceman AM. Gestational weight gain. Am J Obstet Gynecol. 2017;217(6):642–51. doi: 10.1016/j.ajog.2017.05.040 28549978 PMC5701873

[9] Institute of Medicine (US) and National Research Council (US) Committee to Reexamine IOM Pregnancy Weight Guidelines. Weight gain during pregnancy: reexamining the guidelines. Rasmussen KM, Yaktine AL, ed. Washington (DC): National Academies Press (US); 2009. http://www.ncbi.nlm.nih.gov/books/NBK32813/20669500

[10] Thompson AM, Thompson JA. An evaluation of whether a gestational weight gain of 5 to 9 kg for obese women optimizes maternal and neonatal health risks. BMC Pregnancy Childbirth. 2019;19(1):126. doi: 10.1186/s12884-019-2273-z 30975086 PMC6460820

[11] Haugen M, Brantsæter AL, Winkvist A, Lissner L, Alexander J, Oftedal B, et al. Associations of pre-pregnancy body mass index and gestational weight gain with pregnancy outcome and postpartum weight retention: a prospective observational cohort study. BMC Preg Childbirth. 2014;14:201. doi: 10.1186/1471-2393-14-201 24917037 PMC4062904

[12] Singh P, Singh KK, Singh P. Maternal health care service utilization among young married women in India, 1992-2016: trends and determinants. BMC Preg Childbirth. 2021;21(1):122. doi: 10.1186/s12884-021-03607-w 33568078 PMC7877063

[13] Athar U, Daud NUA, Khan WA, Khalid A, Gill SI. Caught between external pressures and internal battles: psychosocial factors affecting gestational weight gain – a scoping review. Cureus. 2021. https://www.cureus.com/articles/51612-caught-between-external-pressures-and-internal-battles-psychosocial-factors-affecting-gestational-weight-gain---a-scoping-review 10.7759/cureus.13487 PMC7989722 33777574

[14] Plante A-S, Lemieux S, Labrecque M, Morisset A-S. Relationship between psychosocial factors, dietary intake and gestational weight gain: a narrative review. J Obstet Gynaecol Can. 2019;41(4):495–504. doi: 10.1016/j.jogc.2018.02.023 30393057

[15] Teede HJ, Bailey C, Moran LJ, Bahri Khomami M, Enticott J, Ranasinha S, et al. Association of antenatal diet and physical activity-based interventions with gestational weight gain and pregnancy outcomes: a systematic review and meta-analysis. JAMA Intern Med. 2022;182(2):106–14. doi: 10.1001/jamainternmed.2021.6373 34928300 PMC8689430

[16] Weeks WB, Chang JE, Pagán JA, Lumpkin J, Michael D, Salcido S, et al. Rural-urban disparities in health outcomes, clinical care, health behaviors, and social determinants of health and an action-oriented, dynamic tool for visualizing them. PLOS Glob Public Health. 2023;3(10):e0002420. doi: 10.1371/journal.pgph.0002420 37788228 PMC10547156

[17] Chi H, Jung S, Subramanian SV, Kim R. Socioeconomic and geographic inequalities in antenatal and postnatal care components in India, 2016-2021. Sci Rep. 2024;14(1):10221. 38702357 10.1038/s41598-024-59981-w PMC11068794

[18] Srivastava S, Muhammad T. Rural-urban differences in food insecurity and associated cognitive impairment among older adults: findings from a nationally representative survey. BMC Geriatr. 2022;22(1):287. 35387591 10.1186/s12877-022-02984-x PMC8985064

[19] Meliko MO, Mossy ER, Ngaiwi ME. Food accessibility measurements amongst rural and urban informal dwellers in Buea Municipality. J Agricul Food Res. 2023;12:100606.

[20] Arora P, Tamber Aeri B. Gestational weight gain among healthy pregnant women from Asia in comparison with Institute of Medicine (IOM) guidelines-2009: a systematic review. J Preg. 2019;2019:1–10. 10.1155/2019/3849596 PMC6421048 30941218

[21] Hamal M, Dieleman M, De Brouwere V, de Cock Buning T. Social determinants of maternal health: a scoping review of factors influencing maternal mortality and maternal health service use in India. Public Health Rev. 2020;41:13. doi: 10.1186/s40985-020-00125-6 32514389 PMC7265229

[22] Vijayalaxmi KG, Urooj A. Impact of socio-economic status on nutritional status of pregnant women and pregnancy outcome. Indian J Nutrit Dietetics. 2009;46(2):50–8.

[23] National portal of India. India at a glance. Accessed 2024 November 18. https://www.india.gov.in/content/rural-indian

[24] Balk D, Montgomery MR, Engin H, Lin N, Major E, Jones B. Urbanization in India: population and urban classification grids for 2011. Data. 2019;4(1):35. 37424897 10.3390/data4010035 PMC10327898

[25] Cohen J. Statistical power analysis for the behavioral sciences. Routledge; 1988. https://www.taylorfrancis.com/books/9781134742707

[26] Wermuth P. Participant recruitment, screening, and enrollment. In: Principles and practice of clinical trials. Springer International Publishing; 2022. 257–78. doi: 10.1007/978-3-319-52636-2_38

[27] Media Research Users Council. Socio-economic classification-2011 the new SEC system. The Market Research Society of India. 2011. https://www.mruc.net/uploads/posts/b17695616c422ec8d9dadafc1c3eec26.pdf

[28] Gilmore LA, Redman LM. Weight gain in pregnancy and application of the 2009 IOM guidelines: toward a uniform approach: approaches estimating GWG. Obesity (Silver Spring). 2015;23(3):507–11. doi: 10.1002/oby.20951 25521748 PMC4340812

[29] Pan WH, Yeh WT. How to define obesity? Evidence-based multiple action points for public awareness, screening, and treatment: an extension of Asian-Pacific recommendations. Asia Pac J Clin Nutr. 2008;17(3):370–4. 18818155

[30] Chasan-Taber L, Schmidt MD, Roberts DE, Hosmer D, Markenson G, Freedson PS. Development and validation of a pregnancy physical activity questionnaire. Med Sci Sports Exerc. 2004;36(10):1750–60. doi: 10.1249/01.mss.0000142303.49306.0d 15595297

[31] Chowdhury Salian S, Singh J. Reliability and validity of the Indian version of the pregnancy physical activity questionnaire (PPAQ). Zenodo. 2017. Accessed 2019 July 25. https://zenodo.org/record/437966

[32] Buysse DJ, Reynolds CF 3rd, Monk TH, Berman SR, Kupfer DJ. The Pittsburgh sleep quality index: a new instrument for psychiatric practice and research. Psychiatry Res. 1989;28(2):193–213. doi: 10.1016/0165-1781(89)90047-4 2748771

[33] Venugopal L, Rajendran P, V P. A study on assessment of sleep quality in south Indian pregnant women. Int J Res Med Sci. 2018;6(10):3197.

[34] Manzar MD, Moiz JA, Zannat W, Spence DW, Pandi-Perumal SR, Ahmed S. BaHammam, et al. Validity of the Pittsburgh sleep quality index in Indian university students. Oman Med J. 2015;30(3):193–202. doi: 10.5001/omj.2015.41 26171126 PMC4459159

[35] Lobel M, Cannella DL, Graham JE, DeVincent C, Schneider J, Meyer BA. Pregnancy-specific stress, prenatal health behaviors, and birth outcomes. Health Psychol. 2008;27(5):604–15. doi: 10.1037/a0013242 18823187

[36] Chiplonkar SA. Trends in nutrient intakes of Indian adults: computerized diet analysis (CDiet) of cross-sectional surveys between 1998 and 2015. CNF. 2021;17(4):423–32.

[37] ICMR-NIN. Recommended dietary allowances and estimated average requirements nutrient requirements for Indians - 2020. Hyderabad: ICMR-National Institute of Nutrition; 2020. https://www.nin.res.in/rdabook/brief_note.pdf

[38] Parent MC. Handling item-level missing data: simpler is just as good. Counsel Psychol. 2013;41(4):568–600.

[39] Martínez-Hortelano JA, Cavero-Redondo I, Álvarez-Bueno C, Garrido-Miguel M, Soriano-Cano A, Martínez-Vizcaíno V. Monitoring gestational weight gain and prepregnancy BMI using the 2009 IOM guidelines in the global population: a systematic review and meta-analysis. BMC Preg Childbirth. 2020;20(1):649. doi: 10.1186/s12884-020-03335-7 33109112 PMC7590483

[40] Dai Z, Li M, Rui L, Sun X, Pang X, Zhou L, et al. Evaluation of pre-pregnancy weight and gestational weight gain among urban and rural women from southwestern China. Wei Sheng Yan Jiu. 2014;43(4):546–9. 25199279

[41] Emery Tavernier RL, McCoy MB, McCarty CA, Mason SM. Trends in maternal weight disparities: statewide differences in rural and urban minnesota residents from 2012 to 2019. Womens Health Issues. 2023;33(6):636–42. doi: 10.1016/j.whi.2023.07.001 37544860 PMC10838365

[42] Gallagher A, Liu J, Probst JC, Martin AB, Hall JW. Maternal obesity and gestational weight gain in rural versus urban dwelling women in South Carolina. J Rural Health. 2013;29(1):1–11. doi: 10.1111/j.1748-0361.2012.00421.x 23289649

[43] Rodrigues PL, de Oliveira LC, Brito A dos S, Kac G. Determinant factors of insufficient and excessive gestational weight gain and maternal-child adverse outcomes. Nutrition. 2010;26(6):617–23. doi: 10.1016/j.nut.2009.06.025 19944566

[44] Daemers DOA, Wijnen HAA, van Limbeek EBM, Budé LM, de Vries RG. Patterns of gestational weight gain in healthy, low-risk pregnant women without co-morbidities. Midwifery. 2013;29(5):535–41. doi: 10.1016/j.midw.2012.04.012 23103320

[45] Arora P, Aeri BT. High pre-pregnancy body mass index and gestational weight gain among women belonging to upper SES from Delhi, India. Eur J Obstet Gynecol Reprod Biol X. 2023;20:100258. doi: 10.1016/j.eurox.2023.100258 37942027 PMC10628650

[46] Abdelwahab M, Voest JA, de Metz TD, Hughes BL, Grobman WA, et al. Gestational weight gain and neonatal biometry during the COVID-19 pandemic: a multicenter observational cohort. Am J Perinatol. 2024. 10.1055/a-2335-2480 PMC11666801 38810962

[47] Harville EW, Kracht CL, Cohen NL, Sutton EF, Kebbe M, Redman LM. Trends in gestational weight gain in Louisiana, March 2019 to March 2022. JAMA Netw Open. 2023;6(8):e2331277. doi: 10.1001/jamanetworkopen.2023.31277 PMC10466167 37642960

[48] Taylor K, Ferreira DLS, West J, Yang T, Caputo M, Lawlor DA. Differences in pregnancy metabolic profiles and their determinants between white European and South Asian women: findings from the born in Bradford Cohort. Metabolites. 2019;9(9):190. doi: 10.3390/metabo9090190 31540515 PMC6780545

[49] Sommer C, Jenum AK, Waage CW, Mørkrid K, Sletner L, Birkeland KI. Ethnic differences in BMI, subcutaneous fat, and serum leptin levels during and after pregnancy and risk of gestational diabetes. Eur J Endocrinol. 2015;172(6):649–56. doi: 10.1530/EJE-15-0060 25740849

[50] ACOG. ACOG Committee opinion: physical activity and exercise during pregnancy and the postpartum period. American Coll Obstetrics Gynecol. 2020;135(4):178–88. 10.1097/AOG.0000000000003772 32217980

[51] Ruifrok AE, Althuizen E, Oostdam N, van Mechelen W, Mol BW, de Groot CJM, et al. The relationship of objectively measured physical activity and sedentary behaviour with gestational weight gain and birth weight. J Pregnancy. 2014;2014:567379. doi: 10.1155/2014/567379 25309754 PMC4189770

[52] Hamann V, Deruelle P, Enaux C, Deguen S, Kihal-Talantikite W. Physical activity and gestational weight gain: a systematic review of observational studies. BMC Public Health. 2022;22(1):1951. 36271388 10.1186/s12889-022-14324-0 PMC9585865

[53] Guilloty NI, Soto R, Anzalota L, Rosario Z, Cordero JF, Palacios C. Diet, Pre-pregnancy BMI, and gestational weight gain in Puerto rican women. Matern Child Health J. 2015;19(11):2453–61. doi: 10.1007/s10995-015-1764-4 26100133 PMC4596788

[54] Hirko KA, Comstock SS, Strakovsky RS, Kerver JM. Diet during pregnancy and gestational weight gain in a michigan pregnancy cohort. Curr Dev Nutr. 2020;4(8):nzaa121. doi: 10.1093/cdn/nzaa121 32793851 PMC7413979

[55] Rugină C, Mărginean CO, Meliţ LE, Giga DV, Modi V, Mărginean C. Relationships between excessive gestational weight gain and energy and macronutrient intake in pregnant women. J Int Med Res. 2020;48(8). doi: 10.1177/0300060520933808 32776838 PMC7418251

[56] Shin D, Bianchi L, Chung H, Weatherspoon L, Song WO. Is gestational weight gain associated with diet quality during pregnancy?. Matern Child Health J. 2014;18(6):1433–43. doi: 10.1007/s10995-013-1383-x 24162550

[57] Hawkins MS, Pokutnaya DY, Bodnar LM, Levine MD, Buysse DJ, Davis EM. The association between multidimensional sleep health and gestational weight gain. Paediatric Perinatal Epidemiol. 2023. doi: ppe.13004 10.1111/ppe.13004 PMC10543452 37641423

[58] Hill C, Lipsky LM, Betts GM, Siega-Riz AM, Nansel TR. A prospective study of the relationship of sleep quality and duration with gestational weight gain and fat gain. J Women’s Health. 2021;30(3):405–11. 10.1089/jwh.2020.8306 PMC7957376 32945728

[59] Kominiarek MA, Grobman W, Adam E, Buss C, Culhane J, Entringer S. Stress during pregnancy and gestational weight gain. J Perinatol. 2018;38(5):462–7. 29379158 10.1038/s41372-018-0051-9 PMC5999529

[60] Smith SM, Vale WW. The role of the hypothalamic-pituitary-adrenal axis in neuroendocrine responses to stress. Dialogues Clin Neurosci. 2006;8(4):383–95. doi: 10.31887/DCNS.2006.8.4/ssmith 17290797 PMC3181830

[61] Cheng TS, Ali N, Elbarazi I, Al‐Rifai RH, Al‐Maskari F, Loney T. Sociodemographic determinants of prepregnancy body mass index and gestational weight gain: The Mutaba’ah study. Obesity Sci Practice. 2022;8(3):308–19. 10.1002/osp4.573 PMC9159565 35664246

[62] Paulino DSDM, Surita FG, Peres GB, Nascimento SLD, Morais SS. Association between parity, pre-pregnancy body mass index and gestational weight gain. J Maternal-Fetal Neonatal Med. 2016;29(6):880–4. 10.3109/14767058.2015.1021674 25758613

[63] Chiba T, Ebina S, Kashiwakura I. Influence of maternal body mass index on gestational weight gain and birth weight: a comparison of parity. Exp Ther Med. 2013;6(2):293–8. doi: 10.3892/etm.2013.1167 24137177 PMC3786819

[64] Bookari K, Yeatman H, Williamson M. Informing nutrition care in the antenatal period: pregnant women’s experiences and need for support. BioMed Res Int. 2017;2017:1–16. 10.1155/2017/4856527 PMC5584352 28890896

[65] Ouédraogo CT, Wessells KR, Young RR, Faye MT, Hess SY. Prevalence and determinants of gestational weight gain among pregnant women in Niger. Matern Child Nutr. 2020;16(1):e12887. doi: 10.1111/mcn.12887 31568674 PMC7038899

[66] Cheney K, Berkemeier S, Sim KA, Gordon A, Black K. Prevalence and predictors of early gestational weight gain associated with obesity risk in a diverse Australian antenatal population: a cross-sectional study. BMC Preg Childbirth. 2017;17(1):296. doi: 10.1186/s12884-017-1482-6 28882122 PMC5590236

[67] Krukowski RA, West DS, DiCarlo M, Shankar K, Cleves MA, Saylors ME, et al. Are early first trimester weights valid proxies for preconception weight?. BMC Preg Childbirth. 2016;16(1):357. doi: 10.1186/s12884-016-1159-6 27871260 PMC5117552

